# MicroRNAs as Potential Predictors of Response to CDK4/6 Inhibitor Treatment

**DOI:** 10.3390/cancers13164114

**Published:** 2021-08-16

**Authors:** Angeliki Andrikopoulou, Almog Shalit, Eleni Zografos, Konstantinos Koutsoukos, Anna-Maria Korakiti, Michalis Liontos, Meletios-Athanasios Dimopoulos, Flora Zagouri

**Affiliations:** 1 Department of Clinical Therapeutics, Alexandra Hospital, Medical School, 11528 Athens, Greece; aggandrikop@med.uoa.gr (A.A.); elzogra@med.uoa.gr (E.Z.); koutsoukos.k@gmail.com (K.K.); anmakor@med.uoa.gr (A.-M.K.); mlionto@med.uoa.gr (M.L.); mdimop@med.uoa.gr (M.-A.D.); 2 Medical School, National and Kapodistrian University of Athens, 80 Vasilissis Sofias Avenue, 11528 Athens, Greece; almog_98@hotmail.com

**Keywords:** microRNAs, biomarker, CDK4/6 inhibitors, resistance, breast cancer

## Abstract

**Simple Summary:**

MicroRNAs are endogenous non-coding 20–22 nucleotide long RNAs that play a fundamental role in the post-transcriptional control of gene expression. Consequently, microRNAs are involved in multiple biological processes of cancer and could be used as biomarkers with prognostic and predictive significance. Cyclin-dependent kinase 4/6 (CDK4/6) inhibitors have become a mainstay of treatment for patients with advanced hormone receptor-positive (HR) breast cancer. Despite the initial high response rates, approximately 10% of patients demonstrate primary resistance to CDK4/6 inhibitors while acquired resistance is almost inevitable. Considering the fundamental role of miRNAs in tumorigenesis, we aimed to explore the potential involvement of microRNAs in response to CDK4/6 inhibition in solid tumors. A number of microRNAs were shown to confer resistance or sensitivity to CDK4/6 inhibitors in preclinical studies, although this remains to be proved in human studies.

**Abstract:**

Cyclin-dependent kinase 4/6 (CDK4/6) inhibitors have emerged as novel treatment options in the management of advanced or metastatic breast cancer. MicroRNAs are endogenous non-coding 19–22-nucleotide-long RNAs that regulate gene expression in development and tumorigenesis. Herein, we systematically review all microRNAs associated with response to CDK4/6 inhibitors in solid tumors and hematological malignancies. Eligible articles were identified by a search of the MEDLINE and ClinicalTrials.gov databases for the period up to1 January 2021; the algorithm consisted of a predefined combination of the words “microRNAs”, “cancer” and “CDK 4/6 inhibitors”. Overall, 15 studies were retrieved. Six microRNAs (miR-126, miR-326, miR3613-3p, miR-29b-3p, miR-497 and miR-17-92) were associated with sensitivity to CDK4/6 inhibitors. Conversely, six microRNAs (miR-193b, miR-432-5p, miR-200a, miR-223, Let-7a and miR-21) conferred resistance to treatment with CDK4/6 inhibitors. An additional number of microRNAs (miR-124a, miR9, miR200b and miR-106b) were shown to mediate cellular response to CDK4/6 inhibitors without affecting sensitivity to treatment. Collectively, our review provides evidence that microRNAs could serve as predictive biomarkers for treatment with CDK4/6 inhibitors. Moreover, microRNA-targeted therapy could potentially maximize sensitivity to CDK4/6 inhibition.

## 1. Introduction

Cyclin-dependent kinases 4 (CDK4) and 6 (CDK6) are crucial mediators of cell cycle progression through G1/S checkpoint regulation. CDK4 and 6 form a complex with Cyclin D1, which catalyzes the phosphorylation of the retinoblastoma protein (RB) [1]. Phosphorylated Rb disassociates from E2F transcription factors and enables the expression of E2F-responsive genes, that are necessary for cell cycle progression. Recently, CDK4/6 inhibitors, palbociclib (PD0332991), ribociblib (LEE011) and abemaciclib (LY2835219), have emerged as new therapeutic options that target the above-mentioned signaling pathway. Specifically, CDK4/6 inhibitors reinstate the Rb-regulated suppression of cell division [1]. Palbociclib was the first CDK4/6 inhibitor to receive US Food and Drug Association (FDA) approval in postmenopausal hormone receptor-positive HR (+), epidermal growth factor receptor 2-negative HER (−) advanced breast cancer, based on the results of the PALOMA-2 Phase III trial [2]. Subsequently, ribociblib and abemaciclib were also approved in combination with endocrine therapy for HR (+), HER2 (−) advanced breast cancer [3,4,5]. Currently, CDK4/6 inhibitors are under investigation as promising therapeutic agents in a wide array of malignancies, although still at a preclinical or early clinical stage [6]. However, mechanisms of de novo or acquired resistance to CDK4/6 inhibitors were reported [7]. Loss of Rb, p16 overexpression or upregulation of the Cyclin E1 gene (CCNE1) were identified as potential mechanisms of resistance [7,8]. Approximately 10% of patients will exhibit primary resistance to CDK4/6 inhibitors, while an increasing number of patients will eventually fail to respond to treatment [7]. Therefore, there is an increasing need for biomarkers to identify non-responders and personalize treatment.

MicroRNAs are endogenous, non-coding 19–22-nucleotide-long RNAs that mediate a posttranscriptional negative regulation of gene expression [9]. The active miRNAs emerge from larger 60–110 nucleotide precursor transcripts that are cleaved by endoribonuclease Dicer. Complementary pairing of miRNA and the 3′ untranslated region (3′ UTR) of their target mRNA leads to mRNA degradation or inhibition of translation. A single miRNA molecule can target multiple mRNAs and, conversely, one mRNA can be the target of multiple miRNAs [9]. Consequently, miRNAs regulate multiple cellular processes, including cell proliferation, migration and apoptosis [10]. Deregulated expression of miRNAs is frequently linked to tumor progression. miRNAs can function as endogenous suppressors of target genes (e.g., miR-34, let-7, miR 200 family), or as oncogenes (e.g., miR-155, miR-17-5p, miR-21) [11,12,13]. All these miRNAs that are implicated in cancer development are known as “oncomirs” [14]. Indeed, miRNAs serve as key regulators of the genome by modulating up to one third of all cellular transcripts [9]. Consequently, these regulatory elements could be exploited as diagnostic, prognostic or predictive biomarkers, or even as therapeutic targets to suppress carcinogenesis.

Given the significance of microRNAs in the regulation of cancer-related pathways, it could be speculated that miRNAs may also play an essential role in response to CDK4/6 inhibitors. Intense research in this field has proposed the implication of miRNAs in observed resistance or sensitivity to treatment. The expression profile of miRNAs in tumor cells could therefore be utilized as a predictive biomarker to guide clinicians in their treatment decisions. Herein, we systematically review all miRNAs that were identified as mediators of response to CDK4/6 inhibitors.

## 2. Materials and Methods

### 2.1. Search Strategy and Eligibility of Studies

This systematic review was conducted in accordance with the PRISMA Statement Guidelines [15]. Eligible studies were sought in the MEDLINE bibliographical database and ClinicalTrials.gov for the period up to 1 January 2021, using the following search algorithm (carcinoma OR carcinomas OR cancer OR cancers OR neoplasm OR neoplasms) AND (microRNA[tiab] OR miR[tiab] OR miRNA[tiab] OR microRNAs[tiab] OR miRs[tiab] OR miRNAs[tiab]) AND (cdk4/6 inhibitors[tiab] OR ribociclib[tiab] OR palbociclib[tiab] OR abemaciclib[tiab] OR PD0332991 OR LY2835219 OR LEE011). Language restrictions were not applied. In order to maximize the amount of synthesized information, we systematically examined the reference lists of the articles retrieved for potentially eligible papers. In cases where overlapping publications emerging from the same study were identified, the larger size study was included.

Eligible articles included all studies exploring the association between microRNAs and response to treatment with CDK4/6 inhibitors in solid tumors. All prospective and retrospective studies, as well as case reports, were considered eligible for this systematic review. Studies evaluating the association of microRNAs with endogenous CDK4/6 inhibitors (e.g., p27Kip1, p21Cip1, CDK inhibitor 2B, CDK inhibitor 1B, PTEN) were excluded from our analysis [16,17,18,19,20,21,22,23,24,25,26,27,28,29,30,31,32,33,34,35,36]. In addition, studies evaluating CDK inhibitors that inhibit other types of cyclin-dependent kinases (e.g., roscovitin (seliciclib)) [21] or long non-coding RNAs [37] were considered ineligible. In vitro and animal studies were included in this study.

### 2.2. Data Extraction

From each of the eligible studies, the following data were collected: name of miRNA molecule, type of CDK4/6 inhibitor administered, type of malignancy in which its expression was determined, miRNA effect on CDK4/6 inhibition, sample type that was used, detection method for miRNA expression and a reference literature. Two investigators (A.A. and A.S.), working independently, searched the literature and extracted data from each eligible study. Any differences in extracted data were resolved via within-pair consensus.

## 3. Results

**Figure 1:**
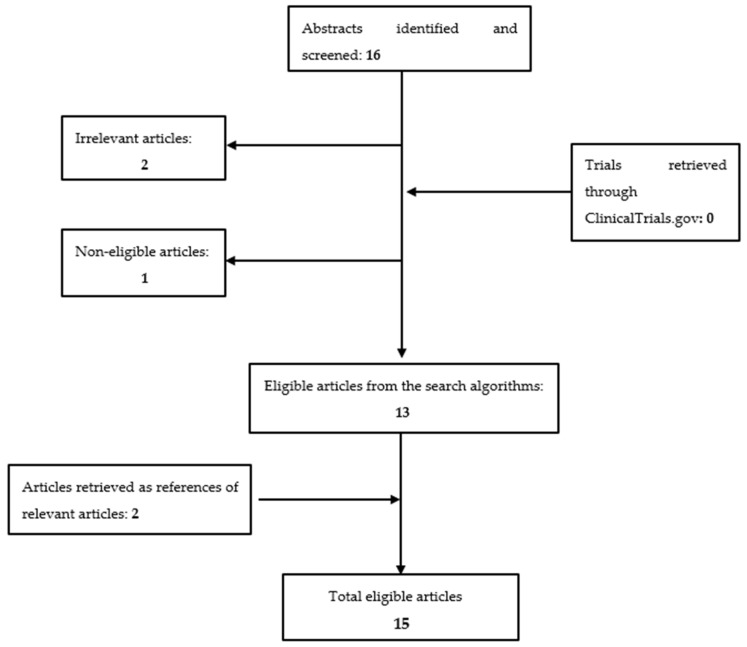
Flowchart presenting the successive steps during the selection of studies.

Overall, 16 articles were identified and screened. After removal of 2 irrelevant studies [38,39] and 1 non-eligible article [37], 13 articles were considered eligible for our study [40,41,42,43,44,45,46,47,48,49,50,51,52]. A thorough search in ClinicalTrials.gov (access date on 1 January 2021) for additional studies retrieved no eligible studies. Two articles retrieved by the search of reference lists of already eligible studies were additionally included [22,53], making a total sum of fifteen studies eligible for inclusion. The successive steps followed during the selection of studies are depicted in Figure 1.

According to our results, six microRNAs (miR-126, miR-326, miR3613-3p, miR-29b-3p, miR-497 and miR-17-92) were implicated with sensitivity to CDK4/6 inhibitors; the first four of the aforementioned molecules were studied in breast cancer cell lines, from the second to the last one in anaplastic large cell lymphoma cell lines and the last one in atypical teratoid rhabdoid tumor cell and glioblastoma stem cell lines (Table 1). Conversely, six microRNAs (miR-193b, miR-432-5p, miR-200a, miR-223, Let-7a and miR-21) conferred resistance to treatment with CDK4/6 inhibitors according to studies performed mainly on cell lines deriving from solid tumors (breast, prostate and metastatic melanoma), with the exception of Let-7a and miR-21, which were upregulated in T-cell acute lymphoblastic leukemia/lymphoma cells (Table 2). An additional number of microRNAs (miR-124a, miR9, miR200b and miR-106b) were shown to mediate cellular response to CDK4/6 inhibitors in acute lymphoblastic leukemia, breast and lung cancer cells, without, however, affecting sensitivity to treatment (Table 3). The following segment contains detailed regulation information for each specific miRNA molecule identified in our study.

### 3.1. MicroRNAs Conferring Sensitivity to CDK4/6 Treatment

miR-126: The study identified four miRNAs, namely miR-9, miR126, miR-181a and miR-326, as the ones with the most profound antiproliferative effect on breast cancer cell lines [43]. Combined miR-126 transfection and CDK4/6 inhibitor ribociclib application resulted in a greater antitumor effect than either agent alone in luminal and HER2-positive breast cancer cell lines (*p* < 0.005) [43]. miR-126 downregulated genes mainly involved in the cell cycle, especially M phase and glycolysis. Collectively, miR126 upregulation sensitized breast cancer cells to CDK4/6 inhibition [43]. Of note, the HER2-positive breast cancer cell line tended to be the most sensitive to miRNA and CDK4/6 inhibitor combination.

miR-326: Ectopic expression of miR-326 exerted a moderate, non-significant antiproliferative effect in breast cancer cell lines (*p* = 0.058) [43]. miR-326 conferred sensitivity to treatment with CDK4/6 inhibitor ribociclib in HER2-positive breast cancer cell lines [43].

miR-3613-3p: Overexpression of miR-3613-3p enhanced sensitivity to the Palbociclib CDK4/6 inhibitor by eliciting senescence [40]. miR-3613-3p was shown to inhibit the growth of TNBC cell lines by targeting SMAD Family Member 2 (SMAD2) and Enhancer of Zeste Homolog 2 (EZH2), two important genes that implicate cell proliferation and cancer growth. Indeed, overexpression of miR3613-3p decreased cell viability of Palbociclib-treated TNBC cell lines, which was partially inversed by the overexpression of SMAD2/EZH2. Overexpression of miR-3613-3p conferred sensitivity to Palbociclib in vivo in murine xenografts carrying miR-3613-3p overexpressing breast tumors. Overall, miR-3613-3p suppressed cell proliferation, migration and clonogenic ability in TNBC cell lines by suspending cells in the G0/G1 phase and efficiently sensitized TNBC cells to CDK4/6 inhibitor treatment.

miR-29b-3p: miR-29b-3p conferred sensitivity to Palbociclib in luminal or HER2-positive cell lines via interfering with the c-myc/miR-29b–3p/CDK6 axis [41]. Upregulation of miR-29b-3p was observed in palbociclib-sensitive breast cancer cell lines after treatment with palbociclib [41]. Overexpression of miR-29b-3p sensitized palbociclib-resistant breast cancer cell lines to palbociclib, while loss of miR-29b-3p induced resistance to palbociclib-sensitive cells. In addition, miR-29b-3p induced cell cycle arrest at the G1 phase, as well as decreased epithelial–mesenchymal transition (EMT) and cell migration. miR-29b-3p exerted its antiproliferative effect via negative regulation of CDK6 in breast cancer cell lines. In addition, c-myc was found to suppress miR29b-3p expression by binding to the promoter region of miR-29b-3p. Therefore, there is an established c-myc/miR-29b–3p/CDK6 axis that regulates palbociclib sensitivity. These findings were replicated in murine models and patient-derived breast cancer xenografts [41].

miR-497: miR-195 and miR-497 were found to be downregulated in NPM-ALK(+) anaplastic large-cell lymphoma (ALCL) patients due to the MIR497HG promoter hypermethylation [42]. Ectopic expression of miR-497, but not miR-195, impaired cell proliferation of NPM-ALK (+) ALCL cells. Transfection of miR-497 reduced the growth of NPM-ALK (+) ALCL cells in vivo in mouse models. miR-497 negatively regulated the expression of cell cycle regulatory genes, such as CCNE1, CDC25A, CDK6 and E2F3, in NPM-ALK (+) ALCL cells [42]. The silencing of these genes suppressed cell growth of ALCL cells in a similar way to miR-497 overexpression. NPM-ALK (+) ALCL cells were sensitive to palbociclib treatment in a CDK6-dependent only manner. Of note, CCNE1, CDK6 and E2F3 expression could serve as a prognostic marker of response to chemotherapy in NPM-ALK (+) ALCL patients and as a marker of response to CDK4/6 inhibition [42].

miR-17-92 (miR-19a, miR-17 and miR-20a): Increased MIR17HG expression, a gene encoding a cluster of six miRNAs (miR-17-92) conferred sensitivity to palbociclib treatment in atypical teratoid rhabdoid tumors (ATRTs) via suppression of the cyclin D1 protein [47]. Susceptibility to CDK4/6 inhibition was confirmed both in vitro in ATRT cell lines and in vivo in an ATRT tumor-bearing mouse. Consistently, ectopic expression of cyclin D1 reduced sensitivity to palbociclib [47]. Atypical teratoid rhabdoid tumors (ATRTs) of the central nervous system are characterized by SMARCB1 loss, a key component of SWI/SNF chromatin remodeling complex. SMARCB1 loss leads to cyclin D1 protein downregulation through activation of MIR17HG gene [47]. Indeed, SMARCB1 associated with the promoter region of MIR17HG in ATRT cell lines, pluripotent human germ cell tumor-derived cells and human liver cancer cells [54,55]. Three of the six miRNAs encoded by the MIR17HG gene (miR-19a, miR-17 and miR-20a) were shown to regulate cyclin D1 protein expression. Indeed, inhibition of MIR17HG, especially miR-17 and miR-19a microRNAs, increased cyclin D1 expression [47]. Conversely, exogenous MIR17HG expression induced cyclin D1 suppression. Overall, cyclin D1 deficiency through upregulation of miR-17-92 microRNAs lead to sensitivity to CDK4/6 inhibitors.

In addition, the miR-17-92 family (including miR-20a and -19a) rendered proneural (PN) glioblastoma (GBM) more susceptible to CDK4/6 inhibition than other subtypes [44]. The miR-17-92 family is upregulated in proneural (PN) glioblastoma (GBM) [44]. This upregulation results from the association of the E2F family of transcription factors with the miR-17-92 promoter. Indeed, the miR-17-92 cluster (miR-20a, -20b, -93, -106a, -130b and -10b) was overexpressed in proneural (PN) GBM stem cell-like (GSC) extracted from patient samples. Glioblastoma (GBM) stem cell-like (GSC) lines were more sensitive to CDK4/6 inhibitors palbociclib and ribociclib than other subtypes. CDK4/6 inhibition induced G1 cell cycle arrest in PN lines while failed to attenuate cell progression in the other GBM cell lines. CDK4/6 inhibitor palbociclib decreased the expression of miR-17-92 family in sensitive PN GSCs by suppressing E2F1 transcription factor.

All microRNAs associated with sensitivity to CDK4/6 inhibition are listed in Table 1.

### 3.2. MicroRNAs Associated with Resistance to CDK4/6 Inhibitors

miR-193b: miR-193b conferred resistance to CDK4/6 inhibition via downregulation of the cyclin D1-encoding gene (CCND1) in prostate cancer [56]. Normally, miR-193b is downregulated via hypermethylation in prostate cancer (PC) cells and patient samples [56]. Expression of miR-193b was inversely associated with cyclin D1-encoding gene (CCND1) expression in PC cell lines and tumor xenografts. Exogenous expression of miR-193b resulted in reduction in CCND1 expression and retinoblastoma (RB) protein phosphorylation. Subsequently, transfection with miR-193b inhibited cell progression to the S and G2/M phases. Low miR-193b expression, thus CCND1 upregulation, rendered prostate cancer cells sensitive to treatment with palbociclib [56]. Conversely, prostate cancer cells expressing high levels of miR-193b levels and low levels of CCND1 were resistant to the CDK4/6 inhibitor palbociclib [56].

miR-432-5p: Estrogen receptor-positive (ER+) cell lines resistant to palbociclib demonstrated increased CDK6 and CCND1 expression along with decreased CDK1 expression [51]. Depletion of CDK6 re-sensitized resistant cells to palbociclib, while CDK6 overexpression increased resistance in parental cells [51]. Coculture of resistant and parental cells resulted in parental cells becoming resistant to palbociclib through extracellular signaling. Overexpression of miR-432-5p was associated with elevated CDK6 expression and resistance to CDK4/6 inhibition with palbociclib or ribociclib. Indeed, miR-432-5p expression was higher in ER (+) tumors with intrinsic or acquired resistance than in tumors sensitive to CDK4/6 inhibition. miR-432-5p upregulated CDK6 expression via suppressing SMAD4 and the TGF-β pathway. These findings were confirmed in breast cancer samples and post-progression biopsies of a CDK4/6 inhibitor-sensitive parotid tumor. The post-progression biopsy exhibited higher miR-432-5p expression and decreased SMAD4 mRNA expression in consistence with resistant tumors. Overall, there is an inverse correlation between the TGF-b pathway and CDK4/6 inhibition. Notably, CDK6 and CCND1 overexpression and miR-432-5p upregulation were all reversible upon palbociclib removal [51].

miR-200a: miR-200a was shown to be epigenetically downregulated during melanoma progression. Indeed, miR-200a expression was significantly decreased in metastatic melanoma [52]. miR-200a negatively regulated CDK6 but not CDK4 expression in metastatic melanoma. Therefore, miR-200a modulated cell cycle progression through the G1/S checkpoint. Downregulation of CDK6 resulted in decreased RB phosphorylation, thus maintaining its repressive effect on cell cycle progression. Moreover, miR-200a downregulation inhibited cell proliferation and colonization. High miR-200a and, consequently, low CDK6 expression were associated with resistance to palbociclib in metastatic melanoma [52]. Inversely, metastatic melanoma cell lines with low miR-200a and high CDK6 expression were more susceptible to CDK4/6 inhibition.

miR-223: Downregulation of miR-223 and the inability to upregulate miR-223 expression was associated with resistance to palbociclib in luminal breast cancer cells [46]. In vivo, miR-223 silenced tumors derived from HER2-positive mammary epithelial cells were resistant to palbociclib treatment. Moreover, palbociclib could efficiently inhibit tumorigenesis only in miR-223 proficient mice that could upregulate miR-223 after treatment. miR-223 expression was increased upon treatment with palbociclib in luminal and HER2 luminal subtypes due to the suppression of the E2F1 transcriptional factor. E2F1 was found to be a strong transcriptional repressor of miR-223 [46]. miR-223 expression was decreased in malignant breast tumors, especially in luminal subtypes, and loss of miR-223 was an early event during mammary transformation [46]. Downregulation of miR-223 expression enhanced cell proliferation and colony formation ability in normal breast epithelial cells though the attenuation of the EGF pathway. Decreased miR-223 levels correlated with reduced overall survival and worse prognosis. Collectively, miR-223 levels could serve as a biomarker to identify responders to CDK4/6 treatment [46].

let-7a and miR-21: This study describes an mTOR–let-7/miR21–CDK6 axis in murine thymic T-cell acute lymphoblastic leukemia/lymphoma tumors (T-ALL/LBL). mTOR inhibition resulted in let-7 and miR21 upregulation and consequent repression of CDK6 [45]. Thymic tumors of mTOR knock-down mice were characterized by upregulation of miR-21 and let-7a miRNAs and undetectable levels of the CDK6 protein. These tumor cells with reduced mTOR activity demonstrated greater resistance to treatment with palbociclib than tumor cells from mTOR wild-type mice [45]. Collectively, genetic or pharmacological downregulation of mTOR resulted in let-7 and miR21 increase and, subsequently, in downregulation of CDK6. Accordingly, simultaneous inhibition of the mTOR pathway and CDK6 activity by palbociclib exerted greater antitumor activity than either treatment alone, both in vitro in human T cell acute lymphoblastic leukemia/lymphoma and in vivo [45].

MicroRNAs that confer resistance to CDK4/6 inhibitors are listed below in Table 2.

**Table 2:** MicroRNAs associated with resistance to CDK4/6 inhibition.

miRNA	Tumor Type	CDK4/6 Inhibitor	miRNA Expression	Mechanism of Action	Biological Sample	Detection Method	References
miR-193b	Prostate cancer	Palbociclib	Epigenetic regulation of expression	Negative regulation of CCND1	Prostate cancer cell lines, fresh frozen tissue, FFPE	qRT-PCR, miRNA assay, IHC	[56]
miR-432-5p	ER+ BC	Palbociclib, Ribociclib	Upregulation	Upregulation of CDK6 via suppression of SMAD4 and the TGF-β pathway	Breast cancer cell lines, FFPE	qRT-PCR, miRNA assay	[51]
miR-200a	Metastatic melanoma	Palbocicib	Epigenetic regulation of expression	Negative regulation of CDK6	Metastatic melanoma cell lines, FFPE	qRT-PCR, NGS	[52]
miR-223	Luminal, HER2+ BC	Palbociclib	Downregulation	Attenuation of the EGF pathway	Breast cancer cell lines, fresh frozen tissue, FFPE, animal models	qRT-PCR, digital droplet PCR	[46]
Let-7a miR-21	Thymic T-cell acute lymphoblastic leukemia/lymphoma	Palbociclib	mTOR suppression mediates upregulation	Downregulation of CDK6	T-ALL/LBL cell lines, animal models	qRT-PCR, microarray analysis	[45]

### 3.3. Others

Some miRNAs were shown to implicate in CDK4/6 inhibitor mechanism of action without clearly resulting in an enhanced or reduced antitumor effect. However, these miRNAs substantially contribute to the antiproliferative role of CDK4/6 inhibitors and are thus listed below (Table 3).

**Table 3:** MicroRNAs that mediate antitumor response to CDK4/6 inhibitors.

miRNA	Tumor Type	CDK4/6 Inhibitor	miRNA Expression	miRNA Mediated Mechanism	Biological Sample	Detection Method	References
miR-124a	ALL	Palbociclib	Epigenetic downregulation	Upregulation of CDK6	ALL-derived cell lines, animal models	miRNA assay, qRT-PCR, ChIP-PCR	[48]
miR9	ALL	Palbociclib	Epigenetic downregulation	Upregulation of CDK6	ALL-derived cell lines	MicroRNA assay	[49]
miR200b	Lung cancer	Palbociclib	Downregulation	Upregulation of CCND1 via upregulation of QKI	Lung adenocarcinoma cell lines, animal models	qRT-PCR, microRNA assay	[50]
miR-106b	ER+ BC	Palbociclib	MCM7 mediated downregulation	Upregulation of p21 and PTEN	Breast cancer cells lines, FFPE	GeneChIP miRNA arrays, qRT-PCR, IHC	[53]

miR-124a: Expression of miR-124a was downregulated in acute lymphoblastic leukemia (ALL) cell lines via hypermethylation of CpG islands [48]. Inversely, upregulation of miR-124a expression was associated with a decrease in cell growth both in vitro and in vivo. Epigenetic downregulation of miR-124a leads to upregulation of CDK6 and activation of the CDK6–Rb oncogenic pathway in ALL cells. Treatment with palbociclib proved to be effective in this CDK6-overexpressing population. Overall, palbociclib inhibited cell proliferation in ALL cells characterized by miR-124a-mediated overexpression of CDK6 [48].

MIR9: CDK4/6 inhibition with palbociclib decreased cell proliferation by targeting the MIR9-mediated upregulation of the CDK6 pathway in ALL cells [49]. The MIR family is epigenetically regulated via promoter hypermethylation in ALL patients [49]. Overexpression of MIR9 resulted in a reduction of CDK6 and phosphorylated-retinoblastoma expression in ALL cells. Consistently, downregulation of MIR9 through hypermethylation induced a significant increase in CDK6 mRNA expression. Of note, patients with non-methylated MIR9 presented with a significantly prolonged disease-free survival (DFS) (*p* < 0.001) and overall survival (OS) (*p* = 0.001). In breast cancer, MIR9 was associated with a non-significant antiproliferative effect [43]. Combination treatment with MIR9 and CDK4/6 inhibitor ribociclib synergized in luminal and HER2-positive breast cancer cell lines, although the interaction failed to reach statistical significance [43].

miR-200b: Endothelial expression of miR-200b was suppressed by more than 50% during lung tumor development. miR-200b was shown to negatively regulate the expression of quaking (QKI), an RNA-binding protein that implicates in endothelial maturation and proliferation. Overexpression of miR-200b inhibited tumor angiogenesis through downregulation of QKI in tumor endothelium [50]. Subsequently, QKI was shown to directly bind to and stabilize cyclin D1 (CCND1) mRNA. This novel miR200b/QKI/CCND1 axis played a role in tumor angiogenesis via the CCND1-mediated endothelial cell cycle progression. Palbociclib inhibited endothelial cell proliferation and tumor angiogenesis by disrupting the CDK4/6–cyclin D complex, thus the miR200b/QKI/CCND1 axis [50]. Palbociclib treatment resulted in a decreased metastatic ability in vivo in tumor-bearing mouse models [50]. Overall, palbociclib inhibited angiogenesis and metastasis via blocking the CDK4/6–cyclin D complex, thus suppressing the function of the miR200b/QKI/CCND1 axis, which regulates endothelial cell growth [50].

miR106b: Palbociclib inhibited tumor growth by downregulating miRNAs, including miR-25 and miR-106b, in ER-positive breast cancer cells [53]. In addition, suppression of the miR106b cluster upon CDK4/6 inhibition was confirmed in breast cancer explants sensitive to CDK4/6 inhibitors [53]. miR106b is regulated via the RB pathway through modulation of MCM7 RB/E2F target gene expression. Downregulation of miR106b cluster resulted in p21Cip1 and PTEN protein upregulation. These findings suggest that CDK4/6 inhibition exerts its antitumor effect via suppressing the expression of the miR106b cluster, thus upregulating p21 and PTEN mRNAs [53]. Indeed, cancer cells expressing the miR106b cluster demonstrated increased cell migration and invasion. Collectively, miR106b expression was associated with a more invasive phenotype of breast cancer through PTEN and the TGF-β pathway.

## 4. Discussion

Overall, we provide preclinical evidence that miRNAs are implicated in the response to CDK4/6 inhibitors. A number of these endogenous molecules seems to confer resistance to CDK4/6 inhibition by altering the expression of downstream target genes, such as CDK6 and Cyclin D1, or downregulating the TGF-β, EGF or mTOR signaling pathways. Conversely, other miRNAs have been associated with sensitivity to CDK4/6 inhibition by interfering with the c-myc/miR-29b-3p/CDK6 axis, eliciting senescence, regulating the expression of cell cycle regulatory genes, such as CCNE1, CDK6 and E2F3, or by reducing cyclin D1 expression. These findings are consistent with the dual role of miRNAs as tumor repressors or tumor promoters [11]. This role is achieved either by directly regulating cell growth and apoptosis or by indirectly targeting other oncogenes or tumor suppressors that modulate cell survival. The vast alteration in the “miRNome” of cancer cells compared with their normal counterparts provides a rationale of increased or decreased response to CDK4/6 inhibitors [12]. The characterization of the miRNA signature of each tumor as a prognostic and predictive biomarker still remains a challenging idea. It should be noted, however, that there is only preclinical evidence of such association and the lack of clinical data is remarkable.

Numerous studies highlighted the link between miRNA expression and chemoresistance. MiR-134, miR-197, miR-490-3P, miR-663 and miR-622 were all diversely expressed in ovarian cancer cells resistant to paclitaxel treatment [57,58,59,60], while the miRNA fingerprint composed of six miRNAs (let-7e, miR-30c, miR-125b, miR-130a and miR-335) could be used as an effective biomarker to identify chemoresistant ovarian cancer cells [61]. miR-27a and miR-451 regulate drug resistance mediated by the MDR1/P-glycoprotein, contributing to an MDR phenotype in cancer cells [62]. miR-134, miR-379 and miR-495 affect the sensitivity of small cell lung cancer cells (SCLC) to chemotherapy, while upregulation of microRNA-451 sensitizes non-small cell lung cancer (NSCLC) cells to cisplatin [63,64]. miRNAs were shown to be involved in chemoresistance of various other neoplasms, including pancreatic cancer [65], hepatocellular carcinoma [66,67], cholangiocarcinoma [68] and esophageal cancer [69,70]. This phenomenon emerges from the robust alteration of various pathways and target genes. Activation of the JAG1-Notch1 signaling pathway [71], EZH2 downregulation [66], Akt signaling pathway upregulation [69] or PTEN downregulation are some of the suggested mechanisms of miRNA-mediated chemoresistance.

Apart from their involvement in response to chemotherapy, miRNAs were shown to mediate the development of resistance to endocrine treatment in luminal breast cancer. Multiple miRNAs modulate estrogen receptor (ER) expression; therefore, they confer resistance to endocrine treatment in breast cancer cells [27,72,73]. miR-221/222, miR-342-3p, miR-873 and Let7b/Let-7i were found to downregulate ERa protein expression and lower ERa levels to serve as a mechanism of resistance [74]. More specifically, luminal breast cancer cells characterized by miR-221/222 overexpression are marked by greater aggressiveness (high Ki67 proliferation index and tumor grade) [74] and higher rates of acquired resistance to selective estrogen receptor downregulator (SERD) fulvestrant [73]. Consistently, overexpression of miR-221/222 rendered breast cancer cells resistant to tamoxifen treatment via targeting p27Kip1 [72]. miRNAs also modulate multiple transcription factors that interact with ERa, including FOXM1, NFκΒ and ERa coactivator nuclear receptor co-activator 3 (NCOA3) [74]. Indeed, miR-30c-5p, miR-30b-5p, miR-182-5p and miR-200b-3p were found to be independent predictors of clinical benefit from endocrine therapy [75]. It should be thus postulated that certain miRNAs may be involved in both CDK4/6 inhibitor and endocrine therapy resistance in luminal breast cancer. Downregulation of ER and activation of the PI3K/Akt/mTOR and CDK4/6/RB pathways are some of the mechanisms of resistance to both endocrine treatment and CDK4/6 inhibitors that could be affected by miRNAs.

Despite the recent establishment of CDK4/6 inhibitors in clinical practice, mechanisms of resistance were already described [7,8]. The amplification of cell cycle regulators, such as p16, CDK6, CCNE1/2, CDK2, CDK7 and E2F transcription factors, plays a key role in de novo and acquired resistance to CDK4/6 inhibitors. Given the strong link between miRNAs and the expression of these cell cycle genes, it could be postulated that antitumor efficacy of CDK4/6 inhibitors could be mitigated by certain miRNAs. However, the interaction of miRNAs with cellular processes, such as cell cycle progression, apoptosis and migration, tends to be more complex, so miRNAs can have both a negative and a positive effect on CDK4/6 inhibition. Since there is a growing attention to identify biomarkers of response to CDK4/6 inhibitors, miRNAs could be used as a valuable alternative.

Another significant aspect of determining the exact relationship between miRNAs and CDK4/6 inhibition is the therapeutic potential they provide. An increasing number of studies utilizes miRNAs to design targeted therapies, such as miRNA inhibitors (antagomirs) or miRNA mimics, to inverse their effect on carcinogenesis [76,77,78,79,80]. NOV340/miR-34a is a novel liposome miR-34a-containing particle that was tested in murine models bearing liver tumors [80]. This novel agent targeting miR-34a was tested in Phase I trial in solid tumors and hematological malignancies (NCT01829971), although the study was prematurely ended due to immune-related adverse events [81]. Moreover, novel compounds, namely, “antagomirs”, that silence miRNAs in vivo were effective in downregulating miRNA expression in mice [78]. “Antagomirs” could be given as a monotherapy or in combination with conventional chemotherapeutic drugs, e.g., doxorubicin, in order to maximize the antineoplastic effect achieved [77]. Once the interaction between miRNAs and CDK4/6 inhibitors is adequately clarified, new treatment strategies combining miRNA-targeting agents and CDK4/6 inhibitors will become feasible.

Among the limitations of this systematic investigation, it should be noted that the study selection process was essentially driven by the search algorithm, which focused primarily on titles and abstracts of the published literature, in order to provide more relevant results. Additionally, clear heterogeneity was observed in our findings, due to differences in malignancies, isolation protocols, detection methods and sample types (i.e., formalin-fixed paraffin-embedded tissues, freshly frozen tumors, cell lines); thus, estimating the pooled effects by performing a meta-analysis was not a feasible task. Lastly, the number of studies on microRNAs associated with response to CDK4/6 inhibitors is still limited, since we are referring to a relatively new treatment modality, suggesting that further studies should be conducted to verify the abovementioned observations.

## 5. Conclusions

Collectively, we demonstrated that miRNAs mediate the function of CDK4/6 inhibitors and are often associated with drug resistance. We reviewed the existing literature to identify those specific miRNAs that have been linked to response to CDK4/6 inhibitors in solid tumors and hematological malignancies. Further investigations are warranted to enlighten the impact of miRNAs on CDK4/6 inhibition and to exploit this association as a novel therapeutic target, since only preclinical evidence is available until now.

## Figures and Tables

**Table 1:** MicroRNAs associated with sensitivity to CDK4/6 inhibition.

miRNA	Tumor Type	CDK4/6 Inhibitor	miRNA Expression	Mechanism of Action	Biological Sample	Detection Method	References
**miR-126**	Lumina, HER2+ breast cancer	Ribociclib	Upregulation	Downregulation of genes associated with cell cycle, especially M phase and glycolysis	Breast cancer cell lines	MicroRNA assay	[43]
**miR-326**	HER2+ breast cancer	Ribociclib	Upregulation	Negative correlation with multidrug resistance-associated protein (MRP-1)	Breast cancer cell lines	miRNA assay	[43]
**miR-3613-3p**	TNBC	Palbociclib	Upregulation	Downregulation of SMAD2 and EZH2	Fresh frozen tissue, breast cancer cell lines	miRNA assay, qRT-PCR	[40]
**miR-29b-3p**	Luminal, HER2+ BC	Palbociclib	C-myc upregulation	Negative regulation of CDK6	FFPE, breast cancer cell lines	miRNA assay, tissue microarrays for IHC	[41]
**miR-497**	NPM-ALK (+) ALCL	Palbociclib	Downregulation via MIR497HG promoter hypermethylation	Negative regulation of cell cycle genes, including CDK6	NPM-ALK ALCL cell lines, tumor tissue	qRT-PCR	[42]
**miR-17-92**	Atypical teratoid rhabdoid tumors	Palbociclib	SMARCB mediated upregulation	Downregulation of cyclin D1	ATRT cell lines, tumor tissue	qRT-PCR, miRNA assay, tissue microarrays for IHC	[47]
	Proneural GBM	Palbociclib, Ribociclib	E2F-mediated upregulation	Downregulation through E2F transcription factors	Glioblastoma stem cell (GSC) lines	qRT-PCR	[44]

## Data Availability

All data can be found at PubMed MEDLINE DATABASE.
